# Plug-and-fight: engineering plant immunity by domain swapping

**DOI:** 10.1016/j.abiote.2026.100050

**Published:** 2026-04-11

**Authors:** Yachun Su, Tingting Sun, Chuihuai You, Shoujian Zang, Youxiong Que

**Affiliations:** a Key Laboratory of Sugarcane Biology and Genetic Breeding, Ministry of Agriculture and Rural Affairs, National Engineering Research Center for Sugarcane, College of Agriculture, Fujian Agriculture and Forestry University, Fuzhou, 350002, China; b State Key Laboratory of Tropical Crop Breeding, Institute of Tropical Bioscience and Biotechnology, Sanya Research Institute, Chinese Academy of Tropical Agricultural Sciences, Sanya, 572024, China

**Keywords:** R gene mining, Plug-in, Genetic engineering, Broad-spectrum resistance

## Abstract

Potato late blight severely threatens global food security. A recent comprehensive analysis of the NLR gene complement across diverse potato (*Solanum tuberosum*) genomes identified previously undescribed resistance genes. This analysis prompted the design of the “domain plug-in” engineering strategy, which involves swapping functional domains between NLRs to broaden the scope of disease resistance. This approach provides a transformative blueprint for designing crops with durable, broad-spectrum disease resistance.

Plant diseases pose persistent, devastating threats to global food security. A prime example is potato late blight caused by *Phytophthora infestans*, with estimated annual yield losses and management costs of US $10 billion worldwide [1]. Nucleotide-binding leucine-rich repeat proteins (NLRs) play central roles in plant innate immunity, conferring race-specific resistance through direct or indirect recognition of pathogen effectors. However, the durability of this resistance is frequently compromised by the rapid adaptive evolution of pathogen populations, constraining resistance breeding. Conventional breeding in autotetraploid potato (*Solanum tuberosum*), which has four sets of chromosomes, is further impeded by its tetrasomic inheritance (each gene having four alleles) and limited sexual hybridization, complicating the stacking of multiple resistance (R) genes. Breeding of diploid hybrid potato based on inbred lines has emerged as a promising alternative for resistance breeding in potato [2]. Nevertheless, the success of this strategy hinges on effective R gene resources and the development of innovative engineering strategies to achieve durable, broad-spectrum protection [3].

Most R genes belong to the NLR multigene superfamily. NLRs are classified into three ancient, functionally divergent subtypes based on their N-terminal domains: TNLs (with Toll/Interleukin-1 receptor [TIR] domains), CNLs (with coiled-coil domains), and RNLs (with RESISTANCE TO POWDERY MILDEW 8 [RPW8]-like CC domains). Additionally, NLRs can be grouped into Type I and Type II based on phylogenetic branch lengths and allelic or orthologous relationships. Efforts to characterize the NLR repertoire have largely relied on isolated genome assemblies, such as a study in potato [4], which, while informative, cannot capture the full pan-genomic diversity and evolutionary dynamics of this complex gene family.

In a recent groundbreaking study published in *Nature*, Wang et al. constructed a section-wide NLRome comprising all 39,211 NLR genes from the genomes of 31 wild and 21 cultivated potato accessions (representing *Solanum* section *Petota*), establishing a comprehensive resource for elucidating NLR evolution and facilitating R gene discovery [5]. The team achieved reference-level genome assemblies for seven late blight–resistant wild potato species, with an average contig N50 value of 19.7 Mb and benchmarking universal single-copy ortholog (BUSCO) completeness scores of 94.6–98.3%. Phylogenomic analysis revealed asymmetric evolutionary patterns among NLRs. Only 7.2% of NLR clades were classified as type I, but they contained 67.1% of all NLRs, primarily comprising sensor NLRs that recognize rapidly evolving pathogens (Fig. 1A). Type II clades accounted for 92.8% of all NLR clades, but only 32.1% of NLR genes, and were enriched for helper NLRs involved in immune signaling. Most sensor NLRs targeting oomycetes, nematodes, and viruses were type I, whereas those recognizing bacterial and fungal effectors were primarily type II, delineating functional specialization within the plant immune system.

**Fig. 1:**
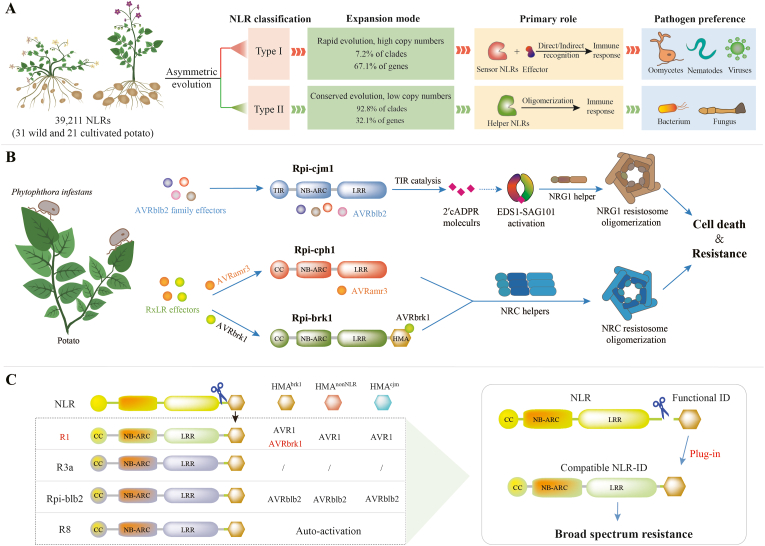
Paradigm for *R* gene mining and *in situ* plug-in strategy for resistance engineering. **A** Classification and evolutionary divergence of potato NLRs. A total of 39,211 NLR proteins from 52 *Solanum* genomes were classified into type I and type II clades, showing asymmetric evolution. Type I clades are rapidly evolving, with increasing copy numbers for sensor genes, encoding proteins that mainly target effectors from oomycetes, nematodes, and viruses. Type II clades are enriched in helper proteins that primarily recognize bacterial and fungal effectors and mediate helper NLR oligomerization to trigger immune responses. **B** Distinct functions of three late-blight resistance proteins. Upon recognition of AVRblb2 family effectors, the TIR domain of Rpi-cjm1 exhibits enzymatic activity and produces the small signaling molecule 2′cADPR, which promotes EDS1-dependent NRG1 helper oligomerization and activates immunity. Rpi-cph1 recognizes the *Phytophthora infestans* effector AVRamr3 and activates NRC helpers to form an NRC resistosome. Rpi-brk1 binds to AVRbrk1 via its HMA domain, thereby activating NRC-dependent defense. **C** Strategy for plug-in resistance engineering. In this modular plug-in design, functional IDs, such as the HMA domain from Rpi-brk1 (HMA^brk1^), are fused to compatible NLRs such as R1, expanding recognition specificity and enabling broad-spectrum resistance. Notably, the HMA^brk1^ domain is only functional when integrated into NLRs of the R1-like NLR clade, but not other NLRs (e.g., R3a, Rpi-blb2, or R8), indicating that effector recognition is not universally transferable. By contrast, two control domains, HMA^cjm^ from a dysfunctional NLR-HMA11 of *S*. *cajamarquense* and HMA^nonNLR^ from a non-NLR protein in *S. tuberosum*, failed to confer AVRbrk1 recognition when fused to R1. The R3a-ID fusion proteins showed no immune responses to any effector examined, and the R8-ID fusion proteins exhibited autoactivation. The effectors AVRbrk1, AVR1, and AVRblb2 are recognized by the resistance proteins Rpi-brk1, R1, and Rpi-blb2, respectively. This approach provides a blueprint for the rational design of durable disease resistance. The figure was created in Adobe Illustrator CS6 software.

Using this resource, Wang et al. cloned three previously unknown late-blight R genes with distinct mechanisms (Fig. 1B) [5]:

i) *Rpi-cph1*, a CNL gene from the wild potato *S. cardiophyllum*, is a functional homolog of *Rpi-amr3*, a broad-spectrum late-blight resistance gene originally cloned from *S. americanum* using single-molecule real-time resistance gene enrichment sequencing (SMRT RenSeq) [6]. In accordance with the NRC helper–dependent immunity model [7], *Rpi-cph1* conferred resistance against five diverse *P. infestans* isolates in transgenic potato plants.
ii) *Rpi-cjm1*, the first TIR-NLR-type late-blight R gene isolated in potato, encodes a protein that recognized four *P. infestans* AVRblb2 effectors and conferred broader-spectrum resistance than that reported for Rpi-blb2 [8]. *Rpi*-*cjm1*-mediated resistance depended on enhanced disease susceptibility 1 (EDS1) and N requirement gene 1 (NRG1). Upon activation, the TIR domain of Rpi-cjm1 exhibited enzymatic activity and produced the signaling molecule 2′-cyclic adenosine diphosphate ribose (2′cADPR), which promotes the oligomerization of helper NLRs such as NRG1, thereby enhancing immune responses. The *Rpi-cjm1* ortholog from *S. sogarandinum* confers resistance to late blight and holds great promise for breeding because it should be possible to introduce it into cultivated potato by crossing.
iii) *Rpi-brk1* encodes an NLR that directly interacted with the *P. infestans* effector AVRbrk1 via its heavy-metal-associated (HMA)-type integrated domain (ID). Seven key residues in this domain were essential for this interaction, as their combined mutations abolished resistance. The role of HMA domains in direct effector recognition was validated in the rice (*Oryza sativa*) Pik NLR system [9], providing a structural paradigm for understanding the Rpi-brk1 mechanism.

The TIR/CC, NB-ARC, and LRR domains are canonical to NLRs, but as exemplified by Rpi-brk1, plant NLRs can harbor additional protein modules known as IDs, which act as molecular decoys by mimicking the host targets of pathogen effectors, thereby enabling the recognition of specific pathogens [3]. Notably, Wang et al. revealed that non-canonical IDs are widespread in the potato NLRome, present in 3.8% (1495 of 39,211) of all NLRs, with the HMA domain being the most common (in 119 NLRs across 46 accessions) [5]. The regions encoding these NLR-embedded IDs displayed stronger signals of adaptive evolution compared to their non-NLR counterparts [5], highlighting their dynamic roles in host–pathogen co-evolution.

The widespread presence and adaptive evolution of IDs in NLRs raised a compelling question: Could these functional modules be harnessed for resistance breeding? Conventional R gene deployment strategies, such as stacking multiple R genes via crossing or co-transformation [10], often face the challenges of linkage drag (the co-inheritance of unwanted traits with a beneficial gene) and laborious selection. To overcome these limitations, Wang and colleagues proposed the “domain plug-in” strategy, a form of functional domain swapping, for resistance engineering [5]. By fusing the HMA domain of Rpi-brk1 to the C terminus of the canonical potato R1 protein, the authors created R1–HMA^brk1^, which recognized its original effector AVR1 and gained the ability to recognize AVRbrk1 (Fig. 1C). This broadened recognition was specific to the R1-like NLR clade. Surprisingly but reasonably, transgenic potatoes expressing *R1–HMA*^*brk1*^ showed strong resistance to *P. infestans* strains secreting AVRbrk1, whereas such resistance was not observed when HMA^brk1^ was fused to NLRs from distinct clades (e.g., R3a, Rpi-blb2, or R8) or when non-functional HMA domains (including the loss-of-function mutant HMA^brk1^-m7) were used. This work demonstrates that non-canonical IDs can be integrated into NLRs to expand the resistance spectrum of existing R genes, when there is compatibility between IDs and NLRs.

This domain-swapping, plug-in strategy combined with genome editing in diploid hybrid potato could facilitate the development of varieties with broad-spectrum resistance and improved agronomic traits, thereby addressing the persistent challenge of late-blight management. Beyond that, mechanistic parallels with other NLR-ID systems point to its broad applicability across diverse plant–pathogen interactions. For example, in the rice blast system, the NLR Pik of rice directly binds to the effector AVR-Pik via its HMA domain [9], employing the same HMA-mediated recognition mechanism. This observation suggests that HMA domains from functionally validated NLRs (e.g., HMA^brk1^) could be swapped into compatible NLR scaffolds in rice to generate new recognition specificities against evolving blast isolates. Likewise, NLR-HMAs are present across multiple Solanaceae species, including the eggplant (*Solanum melongena*) wild relative *S*. *anguivi* and the non-tuber-bearing *S*. *etuberosum* [5]. Therefore, the plug-in strategy might also be extended to other solanaceous crops such as tomato (*Solanum lycopersicum*), pepper (*Capsicum annuum*), and eggplant to engineer resistance against their respective oomycete or fungal pathogens. More broadly, as additional IDs with validated effector-binding functions are identified (e.g., the WRKY domain of resistance to *Ralstonia solanacearum* 1 [RRS1], which recognizes bacterial effectors in *Arabidopsis thaliana* [11]), the plug-in approach could be adapted to deploy these domains across phylogenetically diverse NLRs.

Despite its promise, several challenges must be addressed before the plug-in strategy can be widely employed. Predicting which NLR scaffolds are compatible with which IDs remains difficult, as demonstrated by the failure of HMA^brk1^ to function when fused to R3a or Rpi-blb2. Ensuring that engineered chimeric receptors confer robust resistance without triggering autoimmunity or imposing fitness costs will also require careful optimization. Overcoming these hurdles will depend on deeper structural and functional insights into ID–NLR compatibility and the development of high-throughput screening platforms to identify optimal combinations.

This NLRome resource and domain plug-in engineering strategy hold significant potential for resistance breeding across a wide range of crops. By enabling recognition specificities to be updated in response to pathogen evolution, this modular design offers a transformative pathway toward crops with durable, broad-spectrum disease resistance.

## CRediT authorship contribution statement

**Yachun Su:** Writing – review & editing, Writing – original draft, Visualization, Project administration, Investigation, Formal analysis, Conceptualization. **Tingting Sun:** Visualization, Investigation, Conceptualization. **Chuihuai You:** Visualization, Investigation, Formal analysis. **Shoujian Zang:** Visualization, Investigation, Formal analysis. **Youxiong Que:** Writing – review & editing, Supervision, Project administration, Funding acquisition, Conceptualization.

## Declaration of competing interest

The authors declare that they have no known competing financial interests or personal relationships that could have appeared to influence the work reported in this paper.

## Data Availability

Data sharing is not applicable to this article as no datasets were generated or analyzed during the current study.
